# Complete Genome Sequence of *Mycoplasma hyopneumoniae* Strain KM014, a Clinical Isolate from South Korea

**DOI:** 10.1128/genomeA.01012-17

**Published:** 2017-09-21

**Authors:** Jemin Han, Byung-Sun Park, Dong-Ju Shin, Soo-Yeol Song, Young-Ju Jeong, Nakhyung Lee

**Affiliations:** Technology Institute, KBNP, Inc., Dongan-gu, Anyang, Gyeonggi, South Korea

## Abstract

*Mycoplasma hyopneumoniae* is the etiological agent of swine enzootic pneumonia, resulting in considerable economic losses in the swine industry. A few genome sequences of *M. hyopneumoniae* have been reported to date, implying that additional genome data are needed for further genetic studies. Here, we present the annotated genome sequence of *M. hyopneumoniae* strain KM014.

## GENOME ANNOUNCEMENT

*Mycoplasma hyopneumoniae* is a swine pathogen for enzootic pneumonia and is often a primary agent in the development of porcine respiratory disease complex (PRDC) caused by a mixed infection of several viral and bacterial pathogens (1). Mycoplasma infection in pigs contributes to a mild chronic pneumonia which is characterized by impaired growth performance and depression in feed intake, resulting in considerable economic losses in the swine industry (2). Despite its significant impact on swine production, there is a lack of reliable methods for the control and prevention of this disease. It is partially due to the ineffectiveness of chemotherapy and prophylaxis against this pathogen. Interestingly, it has been reported that *Mycoplasma* species contain some characteristics in their genome sequences in terms of the presence of a strain-specific region, integrative and conjugative elements, genome rearrangements, and the alteration of membrane surface proteins (3, 4). Such genomic features potentially contribute to the differences in pathogenicity and immunity among field isolates.

In light of this information, we report here the full-genome sequence of a clinical isolate, KM014, in South Korea. This strain was isolated from the respiratory tract of a diseased pig in South Korea in 2014. All procedures for isolating and selecting a pure isolate were performed according to a method described earlier (5). Whole-genome sequencing was performed by the combination of PacBio RSII and HiSeq technologies according to the supplier’s protocol. After filtering short reads, a total of 89,180 reads were obtained, covering a total of 673,070,147 bases. The final assembled genome consists of a single circular chromosome of a total of 964,503 bases, with an average G+C content of 28.73%, similar to genomes reported earlier (4). Then, the complete genome was automatically annotated using a Prokka software program, obtaining GBK, GFF3, and SQN files.

The genome sequence predicted a total of 680 putative coding sequences (CDSs). The average size of the putative proteins is 307 amino acids. Seventy-three percent of the putative genes were assigned to a specific functional cluster in the Clusters of Orthologous Groups (COG). The genome also predicted 32 tRNA genes, 3 rRNA genes, and only a single copy of the 16S-23S rRNA operon. The 5S rRNA operon is separate from the 16S-23S rRNA operon. Additionally, this genome revealed several insertions and deletions on some genes, integrative sequences, and unassigned genes. Our analysis indicated that a regional field isolate, strain KM014, contains strain-specific sequences in its genome, as expected.

Together with the genome sequences released earlier, the genome sequence of KM014 presented in this study would provide the potential to understand the biology and pathology of *M. hyopneumoniae* through genome-based research against field isolates.

### Accession number(s).

The genome sequence of *M. hyopneumoniae* strain KM014 has been deposited in GenBank under the accession number CP022714.
